# Neoadjuvant and Adjuvant Chemotherapeutic Strategy of Colorectal Mixed Adeno-Neuroendocrine Carcinomas

**DOI:** 10.7759/cureus.16645

**Published:** 2021-07-26

**Authors:** Anita Michael, Debashis K Nath

**Affiliations:** 1 Internal Medicine, PSG Institute of Medical Sciences and Research, Coimbatore, IND; 2 Internal Medicine, Queen Elizabeth Hospital Kings Lynn, King's Lynn, GBR

**Keywords:** manec, colorectal, neoadjuvant, adjuvant, treatment, management

## Abstract

Mixed adeno-neuroendocrine carcinomas (MANEC) is a rare pathological diagnosis characterized by the presence of both adeno-carcinomatous and neuroendocrine differentiation with each component comprising 30% of the tumor. This literature review is aimed at the extraction of all existing clinical studies and reviews on colorectal MANEC so as to ensure that a suitable chemotherapeutic regimen is chosen to improve survival outcomes and prognosis of the disease. Parallel search strategies were employed to extract past 10 years articles from PubMed, PubMed Central and Google Scholar databases. A total of 30 records consisting of one clinical trial, five retrospective cohort studies, one case control study, one case series, 16 case reports and six review papers were shortlisted. Chemotherapeutic regimens that were administered as an adjuvant and a neoadjuvant therapy were analyzed with their survival outcomes. The overall survival rate of those administered with neoadjuvant and adjuvant therapy can be as high as 57.4% and 69%, respectively. Multiple chemotherapeutic regimens were employed in colorectal MANEC and superiority of one regimen over the other can’t be established. Any drug or combination of drugs that is responsive against either of the MANEC components is found to be effective against the tumor. However, excellent responsiveness has been found with 5-fluorouracil regimens as a neoadjuvant therapy and platinum-based combinations as an adjuvant therapy. XELOX, streptozocin and S1 regimens also prove to be drugs of choice in aggressive and metastasized disease conditions. Our analysis allows for improved chemotherapeutic management of individuals with colorectal MANEC and establishes an increased potential for use of streptozocin therapy in the clinical setting. However, newer drugs like amrubicin require further research prior to describing its efficacy in colorectal MANEC.

## Introduction and background

Mixed adeno-neuroendocrine carcinomas (MANEC) as defined by the World Health Organization (WHO) in 2010, is distinguished by the simultaneous presence of both adeno-carcinomatous (epithelial) and neuroendocrine differentiation, such that each component represents at least 30% of the tumor [1]. Table 1 establishes the classification of MANEC with its histologic appearance as illustrated by La Rosa et al. [2].

**Table 1:** Classification, histological appearance and prognosis of MANEC. MANEC: Mixed adeno-neuroendocrine carcinomas, MANET: Mixed adeno-neuroendocrine tumors, NET-G1: Grade 1 neuroendocrine tumor, NET-G2: Grade 2 neuroendocrine tumor.

Classification of mixed adenocarcinoma-neuroendocrine neoplasms	Composition (histology)	Prognosis
High grade malignant MANEC	Mixed adenomatous / carcinomatous tumor with poorly differentiated neuroendocrine carcinomas.	Worst Prognosis
Intermediate grade malignant MANEC	Mixed adenocarcinoma neuroendocrine tumor that comprises of an exocrine component which is an adenocarcinoma and a neuroendocrine component which is NET-G1 or NET-G2.	Overall Survival (OS) is greater than High-Grade Malignant tumors.
Low-grade malignant MANET	Well differentiated neuroendocrine and exocrine components.	Good Prognosis

Positive immuno-histochemical staining for chromogranin (CgA), synaptophysin (Syn) and CD56 is used to validate the presence of a neuroendocrine component in MANEC. Colorectal neuroendocrine carcinoma (NEC) by itself is a rare pathological diagnosis and constitutes less than 2% of all malignancies of the colon and rectum [3]. The management of colorectal MANEC cannot be attributed to the therapeutic strategy of adenocarcinomas, due to the mixed histological presentation. It is, however, influenced by the type and degree of neuroendocrine differentiation and has a better prognostic score compared to those with neuroendocrine carcinomas [4].

‘National comprehensive cancer network guidelines’ does not provide a specific management strategy for MANEC, however, a complete surgical resection of both the colorectal tumor and metastases followed by chemotherapeutic management (adjuvant) is the widely accepted norm [5]. Neoadjuvant chemotherapy is reported to reduce the risk of metastasis and prolong disease-free survival rates in MANEC [6]. Qiu et al. report that surgical resection with a deliberate adjuvant chemotherapeutic management has a greater survival and a better prognosis [7].

With limited literatures and statistical evidences on colorectal MANEC, it is not possible to estimate the level of similarities to its adenocarcinoma and neuroendocrine counterparts. Moreover, with recognition as a distinct clinical entity, it is imperative that we compile all neoadjuvant and adjuvant chemotherapeutic modalities used and recorded so as to ensure a better management and survival with this rare pathological entity.

## Review

Method and result

Objective and Study Design

Our study design revolves around the extraction of existing neoadjuvant and adjuvant chemotherapeutic strategies to assist in resection and in improving survival. On a secondary perspective, this literature review also hopes to accumulate all observational and case reports to provide with a knowledge of prior experience in chemotherapeutic management of colorectal MANEC.

Retrospective and prospective cohorts, case-control studies, observational studies and case reports on patients diagnosed with colorectal MANEC as well as review papers describing a general and specific management of colorectal MANEC are considered. A search strategy of ‘MANEC’ automatically excludes all articles prior to 10 years, given that the term MANEC was defined as a distinct entity in 2010. However, an additional search filter was placed at past 10-year literatures (2010-2020) to avoid irrelevant search results. Full-text papers were evaluated with language restrictions bounded to English to ensure ease of analysis and tabulation while studies on animal models and meta-analysis were excluded from the study. Pediatric age groups were also excluded from the study due to inconsistent approach of management outline between pediatric and adult (19+) cohorts.

This review has been carried out with a scrupulous attention to the clinical and therapeutic approach and publications that do not justify this approach are excluded. An extensive methodology allowed for a strategic elimination of overlapping literatures and a thorough evaluation of existing literatures that meet with the above criterion.

Search Strategy and Data Collection

Literatures published on and before January 2021 were assessed following the application of a limited number of keywords ‘MANEC’, ‘treatment’ and ‘colorectal’ to ensure that the loss of quality publications is at a minimum. With the availability of limited articles on MANEC, it is imperative that the data procured is maximized. Parallel strategies were used in the evaluation of articles in ‘PubMed’, ‘PubMed Central’ and ‘Google Scholar’ databases. Data collection using the application of MESH keywords was not performed due to a significant decrease in the number of quality articles based on which analysis would then be irrelevant.

Management outlines were not amplified among individuals of varied age (provided they were above 19) and gender. At the same time, tumor advancement, survival, and the type of neoadjuvant and adjuvant chemotherapy is evaluated simultaneously so as to ensure unbiased data collection.

Tumor resection is taken to be a necessity and only chemotherapy administered as a subsidiary treatment before or after surgery is read and described. Hence, margin for error is minimized while maximizing the approach towards this unusual diagnosis.

Search Result and Analysis

An extensive screening and analysis were performed to identify relevant articles in PubMed, PubMed Central and Google Scholar databases. Overlapping or duplicate articles in the three databases were excluded and only those literatures that concede with the objective of the review paper were analyzed and reported. Table 2 provides an insight into the parallel screening process across the three databases on application of inclusion and exclusion criteria.

**Table 2:** Primary screening process of article selection by applying inclusion and exclusion criteria MANEC: Mixed adeno-neuroendocrine carcinomas

Keywords applied	Total records	Records selected
PubMed database		
MANEC	211	174
MANEC, treatment	121	95
MANEC, treatment, colorectal	17	15
PubMed Central (PMC) database		
MANEC	365	321
MANEC, treatment	342	309
MANEC, treatment, colorectal	155	154
Google Scholar database		
MANEC	7590	1470
MANEC, treatment	2150	1230
MANEC, treatment, colorectal	685	524

**Figure 1:**
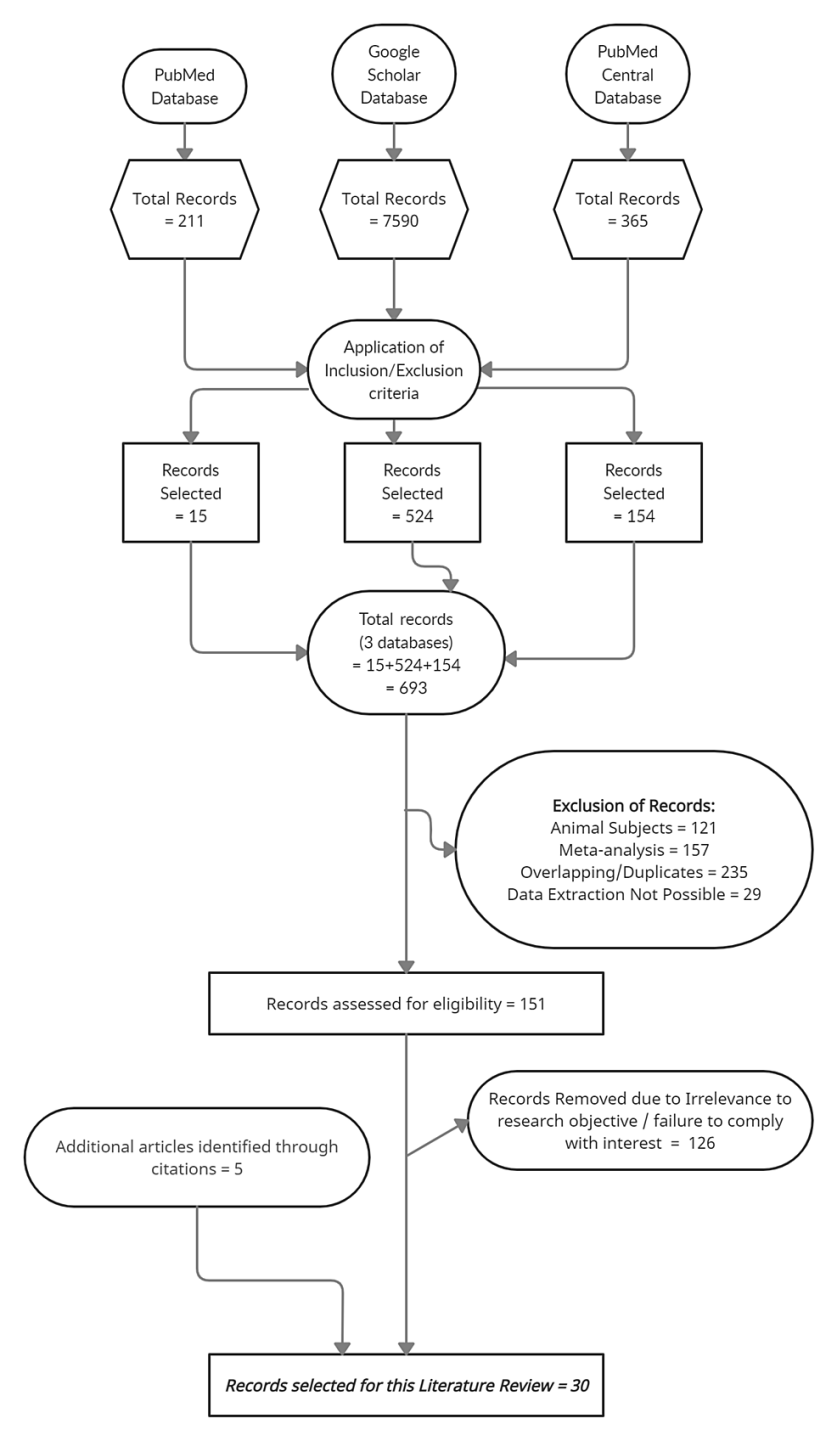
Preferred reporting items for systematic reviews and meta-analyses (PRISMA) flow diagram to summarize the process of screening and literature selection

Primary screening process of article selection yields a total of 693 literatures across three databases. These articles are chosen on the application of basic inclusion and exclusion criteria. A secondary screening process is performed to remove overlapping or duplicate articles, research based on animal models, secondary data analysis (meta-analysis), literatures where data extraction is not possible and those which are irrelevant to the objective of this review. Further, papers which outline a general management of MANEC are also manually chosen and analyzed with a motive to provide potential chemotherapeutic modalities in the neoadjuvant and adjuvant management of colorectal MANEC. Figure 1 summarizes the secondary process of screening and literature selection.

A total of 30 articles are analyzed which include 16 case reports [8-23], one clinical trial [24], five retrospective cohort studies [6, 25-28], one case control study [29], one case series [30] and six reviews [2,3,5,7,31,32]. Table 3 provides a summary of the 16 case reports analyzed in an attempt to throw light onto the individual clinical presentation of patients with MANEC and the chemotherapeutic regimens administered. Tumor staging was based on American Joint Committee on Cancer (AJCC) classification.

**Table 3:** Summary of case reports analyzed in this literature review NC: Neuroendocrine component, CgA: Chromogranin A, Syn: Synaptophysin, a/f: After, N/A: Not applicable, +/-: Positive/Negative, ND: Not described, LAD: Gastric lymphadenectomy, FLOT: Fluorouracil, leucovorin, oxaliplatin and docetaxel, XELOX: Capecitabine and oxaliplatin, TP: Paclitaxel and cisplatin, 5-FU: 5-fluorouracil, FOLFOX: Leucovorin, fluorouracil and oxaliplatin, taTME: Transanal total mesorectal excision, HAI: Hepatic artery infusion, CDDP: Cisplatin, CTP-11: Irinotecan, TACE: Trans arterial chemoembolization.

			Immunohistochemistry (Of NC)						Chemotherapy			
S. No.	Literatures	Age of the individual (years)	Stage of tumor	CD56	CgA	Syn	Ki67	Resection	Neoadjuvant	Adjuvant	Additional chemotherapy (in case of disease progression)	Survival (a/f resection)
1	Li et al. (2020) [8]	55	IV	+	+	+	>90%	Not possible	S1 regimen consisting of titanium silicate (TS1), tegafur, gimeracil, oteracil and potassium combined with cisplatin is prescribed.		N/A	31 Months
2	Oneda et al. (2019) [9]	73	I	+	ND	+	ND	LAD D2 surgery performed	FLOT	FLOT	N/A	Death did not occur
3	Lin et al. (2019) [10]	60	IIIB	+	+	+	>70%	Laparoscopic radical gastrectomy	ND	XELOX	TP therapy following radiofrequency ablation for liver metastasis	Death did not occur
4	Gul-Klein et al. (2018) [11]	65	IV	ND	ND	+	>70%	Rectum resection + atypical liver resection (segment II)	5-FU radio chemotherapy	FOLFOX	Bevacizumab and capecitabine regimen following low progression of bi pulmonary metastases.	17 months
		63	IIIB	ND	ND	+	>90%	Combined laparoscopic and taTME		XELOX	Chemotherapy switched to carboplatin/etoposide following pulmonary metastasis and progressive peripheral neuropathy	12 months
5	Shin et al. (2017) [12]	32	IIIC	ND	+	+	ND	Right hemicolectomy performed	ND	FOLFOX	No recurrence	Death did not occur
6	Tagai et al. (2017) [13]	36	I	+	ND	+	>70%	Right hemicolectomy performed	ND	FOLFOX6 followed by XELOX after 3 cycles	On suspecting bone metastasis with XELOX therapy, XELOX with bevacizumab was given. Streptozocin monotherapy administered on worsening of multiple liver metastases	20 months
7	Silva et al. (2017) [14]	76	I	ND	ND	ND	ND	Endoscopic mucosal resection	ND	N/A	No recurrence/disease progression	Death did not occur
8	Morais et al. (2016) [15]	64	IIIB	+	+	+	>20%	Right colectomy performed	ND	FOLFOX	N/A	5 months
9	Gurzu et al. (2015)[16]	74	IIB	ND	-	+	<20%	Right hemicolectomy with terminal ileum resection	Emergency resection	FOLFOX	No recurrence/disease progression	10 months (Death did not occur)
10	Minaya-Bravo et al. (2015) [17]	66	IIIB	ND	+	+	>20%	Subtotal colectomy due to diverticulosis followed by ureterectomy.	ND	Adjuvant chemotherapy was declined	N/A	Death did not occur
11	Vanacker et al. (2014) [18]	30	I	+	ND	+	75%	Emergency laparotomy and right hemicolectomy performed	Emergency resection	Cisplatin and etoposide followed by high-dose induction chemotherapy (carboplatin, mitoxantrone and cyclophosphamide)	No disease progression (Note: Autologous stem cell transplantation was performed as a part of management strategy)	30 months (Death did not occur)
12	Yamauchi et al. (2014) [19]	41	I	+	+	ND	<2%	Right hemicolectomy performed	ND	No adjuvant chemotherapy (Low-grade malignancy)	No recurrence/Disease Progression	18 months (Death did not occur)
13	Ito et al. (2014) [20]	39	IV	+	+	+	80%	Partial resection of transverse colon followed by hepatectomy	ND	HAI chemotherapy with 5-FU is administered followed by cetuximab + mFOLFOX6 (folinic acid, fluorouracil, and oxaliplatin) + octreotide.	Disease progression necessitated CDDP + CPT11 chemotherapy and TACE	4 months (110 days)
14	Koletsa et al. (2014) [21]	84	IIA	ND	+	+	60%	Resection of sigmoid colon	ND	No adjuvant chemotherapy (Low-grade malignancy)	No recurrence/disease progression	2 years (Death did not occur)
15	Jain et al. (2013) [22]	68	IIIC	ND	+	+	>20%	Right hemicolectomy performed	ND	ND	N/A	Death did not occur
16	Liu et al. (2014) [23]	68	IIA	+	+	+	70%	Intestinal tube (30 cm × 13 cm × 4.5 cm) and appendix on ileocecal valve was resected	ND	ND	No recurrence/disease progression	3 months (Death did not occur)

S1 regimen consisting of titanium silicate, tegafur, gimeracil, oteracil and potassium is used in the management of MANEC in conditions where resection is not possible or indicated. S1 regimen combined with cisplatin is considered to be effective with survival up to 31 months [8].

In cases where resection is indicated, neoadjuvant chemotherapy is given less importance by practitioners as opposed to adjuvant therapy which is seen almost as a necessity to prevent relapse or recurrence. It is not feasible in stage one tumors with low proliferative rates [12] and cases where diagnosis of MANEC is coupled with emergency resection [9,11]. In the advent of administration, an antimetabolite, commonly fluorouracil and its derivatives is prescribed with an objective to inhibit tumor progression or growth prior to resection [9,11].

Stage I MANEC can exhibit high proliferative rates (Ki67 index >70%), progressive metastases and a possible death, hence frequent examination and changes in chemotherapeutic drugs for all stages of tumor are essential to increase tumor response [13]. Pulmonary and hepatic metastases necessitate a switch in chemotherapeutic regimens [10,11]. Streptozocin monotherapy was found to be effective in the treatment of unresponsive hepatic metastases [13]. Autologous stem cell transplantation was also used as a management strategy along with adjuvant chemotherapy in the prevention of disease progression [18].

Patients with stage IV tumors show a survival of up to 17 months [11] following resection and 31 months on administration of S1 regimen [8]. Individuals with stage I tumors can live up to 30 months or more [18], however, the survival depends equally on the rate of proliferation of tumor (Ki67 index) as that of the tumor stage. Table 4 describes the summary of clinical studies analyzed with an insight into the overall survival rate of individuals with colorectal MANEC.

**Table 4:** Summary of clinical studies analyzed in this literature review OS: Overall survival, ORR: Overall response rate, CT: Clinical trial, RC: Retrospective cohort study, CC: Case control study, CS: Case Series, NAC: Neoadjuvant chemotherapy, EP: Etoposide and cisplatin, IP: Cisplatin and paclitaxel, AC: Adjuvant chemotherapy, 5-FU: 5-fluorouracil, ECC: Epirubicin, cisplatin and capecitabine, XELOX: Capecitabine and oxaliplatin, FOLFOX: Leucovorin, fluorouracil and oxaliplatin.

S. No.	Literatures	Study Design	Sample Size	Follow-Up (Months)	Chemotherapy analyzed	OS (%)	ORR (%)
1	Araki et al. (2016) [24]	CT	19	11.5	Amrubicin administration	-	18.8
2	Ma et al. (2020) [6]	RC	69	61	NAC (EP/IP vs. No NAC)	57.4	65
3	Lin et al. (2020) [25]	RC	804	62	AC (comparison between 5-FU, no 5-FU and no AC)	53.2	-
4	Song and Yuan (2019) [26]	RC	131	36	Chemotherapy compared to no chemotherapy	37.68	-
5	van der Veen et al. (2018) [27]	RC	49	60	NAC (between platinum, non-platinum and no NAC) AC (ECC, EP, XELOX and no AC)	39	-
6	Komatsubara et al. (2016) [28]	RC	12	36	AC (5-FU and other AC regimens)	27	-
7	Watanabe et al. (2016) [29]	CC	42	40	AC (FOLFOX, FOLFOX + cetuximab, XELOX, XELOX + bevacizumab)	69	40
8	Dulskas and Pilvelis (2019) [30]	CS	9	17	Chemotherapy, radiotherapy or both administered	55.6	-

The overall survival rate of individuals treated with adjuvant or neoadjuvant chemotherapy is found to be significantly higher than those treated with only resection. An overall survival rate of up to 57.4% and 69% in those administered with neoadjuvant and adjuvant therapy respectively can be seen in individuals with colorectal MANEC. Platinum-based regimen etoposide + cisplatin and cisplatin + paclitaxel (EP/IP) seems promising as a first line drug with a response rate of 65%. A new drug amrubicin is under clinical trials and reports a response rate of 18.8%.

The maximum number of individuals employed in the study stands at 804 with the minimum number at nine. 5-fluorouracil-based compounds, XELOX, FOLFOX and platinum-based combinations particularly etoposide and cisplatin have been studied extensively among the cohort.

Discussion

Multiple treatment options are considered and a variety of regimens have been prescribed in the treatment of colorectal MANEC. No regimen is considered to be superior over the other. Nevertheless, it is important to note that the duration of survival and response to different regimens greatly varies within the population.

S1 Regimen

Doublet chemotherapy consisting of tegafur, gimeracil, oteracil and potassium is a common adjuvant therapy in countries such as Japan. It can also be administered in combination with cisplatin as an effective neoadjuvant therapy [33]. S1 regimen is commonly administered in advanced or stage IV colon cancers and MANEC where resection is not indicated [8,33]. However, it is also seen as a gold standard adjuvant therapy in stage II or III gastric cancers and in hepatic metastases [34]. Anemia, leukopenia and neutropenia are commonly observed adverse drug reactions. However, it is seen as a relatively safer option with an optimal dose reduction following curative resection in stage III gastric cancers. 74.2% of individuals who participated in the study have completed the regimen [35].

A combination regimen of S1 along with cisplatin, paclitaxel, docetaxel and irinotecan is recommended to further improve survival outcomes [32]. While there has been limited study on the use of this regimen in MANEC, a positive response in gastric carcinoma shows potential for its use by targeting the adenocarcinomatous component.

XELOX Regimen

The XELOX regimen uses capecitabine and oxaliplatin as an adjuvant therapy in the treatment of individuals with gastric carcinomas and preferably in those with hepatic metastases. National comprehensive cancer network (NCCN) guidelines recommends its use in individuals following R0-D2 resection in gastric carcinomas [36]. A median overall survival (OS) rate of 19.8 months was observed with decreased incidences of Grade III or IV toxicities. Adverse effects were mild and include nausea, vomiting and diarrhea with occasional cases of paresthesia and peripheral neuropathy [37]. However, the response to XELOX therapy varies among individuals and it is recommended that preoperative treatment regimens and nutritional status of the individual is taken into consideration prior to administration [10].

Fluorouracil-Based Treatment Regimens

The recommended fluorouracil-based treatment regimens for MANEC include the ‘FLOT’ (fluorouracil, oxaliplatin and docetaxel) and ‘FOLFLOX’ (fluorouracil, leucovorin and oxaliplatin) regimens. Fluorouracil-based adjuvant therapy is targeted towards the adenoneuroendocrine component of the tumor [15]. Fluorouracil has an antimetabolic activity, thereby killing and inhibiting the growth of tumor cells. Hence, fluorouracil as a part of the FLOT regimen is also used commonly as a neoadjuvant therapeutic drug.

FLOT neoadjuvant therapy reports an increased overall survival (OS) of up to 50 months and an increased likelihood for the patient to undergo all stages of chemotherapy. Overall response rate (ORR) is considerable and is reported to be up to 55%. While the incidences of leucopenia and neutropenia were less, a few individuals also reported grade III to IV hematological abnormalities and one patient dropped out of neoadjuvant therapy due to acute cerebral infarction [33]. With the use of FOLFOX therapy, duration of FLOT therapy can be reduced with comparable efficacies in stage III colon cancers. Six-year overall survival (OS) stands at 72.9% with the administration of FOLFOX regimen [38].

This review suggests FOLFOX over FLOT in patients with poor performance status due to the presence of significant adverse drug reactions but similar treatment response.

Platinum-Based Combination Regimens

Platinum-based combination regimens which are used as an adjuvant therapy for MANEC use cisplatin or carboplatin in combinations with etoposide, irinotecan and docetaxel. These have been described as TP (cisplatin + docetaxel), CDDP + CPT 11 (cisplatin + irinotecan), EP (cisplatin + etoposide) and carbo/etop (carboplatin + etoposide) regimens. These drugs have been administered in cases with high proliferative rates with increased risks of disease progression [10,11,18,20]. TP therapy can be used as a second line drug in liver metastases and EP therapy has been used in pulmonary metastasis of MANEC [10,11]. Platinum-based combination regimens, however, account for a number of toxicities with reports of renal dysfunction and febrile neutropenia [39] which makes it difficult for the administration of a second or third line chemotherapeutic drug into the clinical setting.

The involvement of neuroendocrine component in MANEC allows for a similar clinical behavior between MANEC and neuroendocrine tumors. Poorly differentiated gastrointestinal neuroendocrine tumors are highly aggressive and it is recommended that these tumors are treated with an EP regimen according to European neuroendocrine tumor society (ENETS) guidelines [36] and a carbo/etop or CDDP + CPT11 regimen according to national comprehensive cancer network (NCCN) guidelines [40]. Nordic guidelines 2014 for diagnosis and treatment of gastroenteropancreatic neuroendocrine neoplasms also recommend an EP or carbo/etop regimen in the treatment of neuroendocrine neoplasms [41].

EP regimen is considered to have a high response rate among individuals with colorectal high-grade metastatic neuroendocrine tumors, however, this is often followed with poor overall outcomes [31]. Hence, Vanacker et al. use autologous stem cell transplantation and high dose induction chemotherapy consisting of carboplatin, mitoxantrone and cyclophosphamide to improve survival outcomes of the individual. It reports that EP regimen alone following resection exhibits a maximum survival rate of not more than 17 months [18].

Platinum-based regimens are recommended if the Ki67 index of the neuroendocrine component is equal to or more than 55%, which otherwise is considered to be ineffective [39]. No specific role is described for systemic adjuvant therapy in grade I and grade II neuroendocrine tumors, however, from a clinical perspective, we recommend its use in grade I and grade II highly proliferative tumors.

Streptozocin Monotherapy

‘The guidelines committee of the Japan neuroendocrine tumor society’ suggests the use of streptozocin, a cytotoxic agent with 5-fluorouracil or doxorubicin for the treatment of grade I and grade II neuroendocrine tumors [42]. It is also considered to be more effective as compared to monotherapy [43]. Tagai et al. report a partial response to streptozocin monotherapy on treatment of colorectal MANEC [13] and is also reported to show a response rate of about 25% in gastrointestinal neuroendocrine tumor patients [44].

Krug et al. report that 3% of individuals presented with renal failure and only 10% reported with grade III or IV toxicities [43]. The common adverse reactions reported with streptozocin therapy were mild and include vomiting, nausea and lethargy [44]. This limited adverse reaction also paves way for the safe administration of a third line chemotherapeutic drug in the setting. While no other reports of streptozocin therapy for the treatment of MANEC can be found, it is recommended for daily practice in pancreaticoduodenal and gastrointestinal neuroendocrine tumors [44], and thus can be safely assumed that it has the potential to be an effective treatment regimen in the management of colorectal MANEC.

Prognosis

A limited number of literatures makes it difficult to determine its prognosis. A median survival rate is estimated to lie between seven to 10 months following diagnosis and prognosis appears to be bleak [11]. However, benign and low grade malignant cases of MANEC show cure without disease progression and recurrence. Submucosal layer involvement of low-grade malignancies, proliferative rate of the tumor and the percentage of neuroendocrine component in the tumor determine its prognosis to some extent although a definite conclusion cannot be made [21].

Limitations

Only full text articles were included for an ease of accessibility and non-English literature was removed to avoid misinterpretation and translational error. This could have underestimated the available clinical data as a result of decreased sample size analysis. Multiple studies on MANEC were performed on animals, however, a significant difference in biological structure and the lack of a control group undermined the statistical power of the study. Pediatric population were excluded due to differences in the presentation of the disease and subsequent treatment regimens. The number of individuals employed in the study was decreased due to rare presentation of the disease. Regimens where chemotherapy was given as an adjuvant or a neoadjuvant to resection were only considered to maintain uniformity of the research analysis. This, however, could have resulted in loss of articles that describe for the use of a potential chemotherapeutic drug in colorectal MANEC management.

## Conclusions

Any chemotherapeutic treatment regimen that is focused towards the adeno-carcinomatous or neuroendocrine component will be partially or completely effective against the tumor subtype. However, neuroendocrine tumors are highly aggressive and it is suggested that an adjuvant therapy focused on the neuroendocrine component of the tumor (for example: platinum-based combinations or streptozocin therapy) is administered as a first-line adjuvant therapy. In the current scenario, fluorouracil-based treatment regimens such as FLOT, FOLFOX, mFOLFOX are commonly employed in the neoadjuvant setting due to the antimetabolic activity of fluorouracil compounds and its high survival rate. FOLFOX is recommended due to decreased risk of adverse effects and similar effectiveness to FLOT. XELOX and platinum-based regimens have been initiated in the adjuvant setting with excellent responsiveness while streptozocin therapy shows great potential as an effective regimen in the management of colorectal MANEC.
